# DOES PAIN PREDICTS FRAILTY AMONG OLDER ADULT AMERICANS: FINDINGS FROM THE NATIONAL HEALTH AND AGING TRENDS STUDY.

**DOI:** 10.1093/geroni/igz038.3194

**Published:** 2019-11-08

**Authors:** Jaspreet k Sodhi, Lin-Na Chou, Soham Al Snih

**Affiliations:** 1 The University of Texas Medical Branch, Galveston, Texas, United States; 2 University of Texas Medical Branch at Galveston, Galveston, Texas, United States

## Abstract

Musculoskeletal pain is highly prevalent among older adults and is one of the common causes of disability. The objective of this study was to examine the effect of pain on becoming frail among American older adults over 6 years of follow-up. We studied 5,229 participants aged ≥ 65 years from the National Health and Aging Trends Study (2011-2017) who were non-frail at baseline. Key variables included pain, socio-demographic characteristics (age, gender, race/ethnicity, marital status, and education), depression, comorbidities, and body mass index. The outcome variable was frailty assessed using the frailty phenotype, defined as meeting three or more of the following criterions: shrinking, weakness, exhaustion, slowness, and low physical activity. General estimation equation model was fitted to test the effect of pain on frailty over time. Prevalence of pain in American older adults was 48.9% at baseline. The prevalence of frailty ranged from 6.7 % at baseline to 7.4 % at wave 6 among those with pain. The odds ratio (OR) of becoming frail overtime was 1.07 (95% CI 1.02 – 1.12) over time. The OR of becoming frail over time as a function of pain was 1.76 (95% CI 1.51-2.05), after controlling for all covariates. Other predictor factors of becoming frail were being ≥ 75 years, having one or more comorbid conditions, and with high depressive symptoms. Participants with higher level of education were less likely to become frail. These findings suggest that early treatment of pain may reduce frailty and improve the quality of life in this population

